# Why should academia care about the Target Product Profile?

**DOI:** 10.1186/s12967-024-05520-0

**Published:** 2024-08-02

**Authors:** Marina Boccardi, Michael Gold, Vijay Mahant, Francesco M. Marincola, Angus Gunn

**Affiliations:** 1 German Center for Neurodegenerative Diseases (DZNE), Rostock-Greifswald site, Rostock, Germany; 2 Department of Psychosomatic Medicine and Psychotherapy, University of Medicine Rostock, Rostock, Germany; 3 AriLex Life Sciences LLC, Deerfield, IL 60015, USA; 4 Renova Therapeutics Inc, Carlsbad, CA USA; 5 Sonata Therapeutics, Watertown, MA USA; 6 UCB, Brussels, Belgium

## Background problem

The attrition rate of biomedical translational research is greater than 90% [1–3]. Translational studies attempt to fill the gap between basic research and the application of its results. Those investigating the early elements of the translational potential do not suffer from methodological weaknesses, because the required study designs and outcome measures are similar to those used in basic research. However, those covering subsequent translational phases, meant to *develop* a potential clinical advance into a concrete and marketable product, require a different approach, characterized by a *forwards*, rather than *backwards*, way of thinking. *Thinking forwards* means producing the data that the stakeholders processing the next development steps need to receive from those who worked at the previous development step along the translational continuum. However, the attitude usually characterizing academic translational studies is mostly based on *thinking backwards.* This entails investigating a topic that was poorly investigated before, and for which researchers can therefore get funds and space to publish the results. The survey of methodologies for developing diagnostics reported in [4] provides reference examples of the feed-forward development flow. Lack of awareness of this key difference in the methodology of translational studies in academic contexts [5] contributes to the enormous attrition rate. A concrete example is encountered while developing clinical guidelines, where extensive literature cannot be used to make decisions, since studies answered “backwards” (e.g., *can* a new biomarker detect a specific disease) rather than “forwards” questions (e.g., *if and how much more accurate* is a new biomarker-based diagnostic procedure compared to the traditional diagnostic procedure, not using that target biomarker); and this happens despite the data available to researchers could actually answer both questions [6].

The industrial pharmaceutical and biotech sector developed more effective techniques to guide translational research and product implementation. Learning from this field may help academic researchers to improve the efficiency of their own translational research, as well as their ability to effectively collaborate with industry.

### Target product profiles: what they are, and why are they important in industry?

A key industrial tool guiding the translational process in industry is the Target Product Profile (TPP) [7, 8]. This tool helps to define a priori, for a specific product or service, what minimal features or parameters are needed, to ensure a reasonable probability of commercial success. To this end, TPPs help to understand the product on its journey to implementation, or to capture poor feasibility early on. They help understand competition and commercialization prospects; to define the value of the asset and detail its development needs as robustly as possible, considering the uncertainties posed by evolving science, regulatory policies, health care systems and geopolitical environments. They help sorting out cost/pricing to the consumer/patients and who will pay (patient, government or insurance company? ). By making development goals and their requirements objective, TPPs also provide a framework that enables data-driven decisions ranging from accelerating investment in a promising asset to abandoning the product if key features are not met. Under this scenario, TPPs can concretely support the implementation of the systematic development frameworks outlined in [4], helping to look *forward* and spell out the part of development meant to meet regulatory or other requirements into clear, consecutive, actionable steps. Given the varied participation of stakeholders along such development (regulators, health technology assessment experts, providers of required technology, decision makers, investors, patients, health payers or providers, etc.), TPPs are a tool to align developers’ and stakeholders’ perspectives into an executable program, improving communication and co-development while minimizing risks, rate of failures and costs.

### Why is this important in academia?

Academia is increasingly engaged in developing therapeutic and diagnostic products, generating novel intellectual property and patents, and for this reason technology transfer offices are increasingly incorporated in academic institutions. However, also activities more traditionally performed by academics entail developing novel ideas from mere theoretical concepts to marketable products. Medical guidelines, new diagnostic or rehabilitation procedures, or any kind of service are equally subject to sound development needs to guarantee acceptability, refund and uptake in clinical contexts, and must equally address multiple requirements and stakeholders’ perspectives. Despite this, academic contexts lack the expertise on commercial, regulatory and other key development aspects, there included a systematic reference framework structuring an efficient workflow: eventual implementation can only be achieved through proper “*feed forward”* processing, i.e., by producing the results that are needed by the next stakeholder for the next development step, and that must therefore be generated with specific methods, as outlined in [4].

To improve under such framework, academia may benefit from collaboration with industry, and a starting approach may consist of importing some of its tools and good practice procedures. The value of TPPs is that they can easily operationalize such development steps and their sequence, and help engage the relevant stakeholders to spell out such consecutive steps for increased commercialization prospects. Moreover, TPPs are devised as living tools, having the flexibility to incorporate new information as development advances and the context evolves. In [9], Cocco and colleagues provide one such example of how TPPs can operationalize development steps to develop diagnostics for infectious diseases. In this number, Ibnidris et al. [10] expand such effort to make the tool even more accessible to academic researchers. This entails clarifying how TPPs are defined and used, and including the “revisions” of existing TPPs, thus attesting their “living” character. Still, this effort should rest on a much wider effort to create awareness on the features and needs of the *feed-forward* translational path, on a greater ability to include different stakeholders from the beginning of product development, and on an increased interest to aspects that will *later* determine whether a product is viable. Without a coordinated action addressing such elements of this wider and *prospective* context, the attrition rate of translational research will keep being disproportionately high.

### Which audience, which hurdles?

The main target audience of this effort is the community of academic translational researchers, who, like most scientists, tend to be “hyper-specialized” to face increasing complexity and competition. This environment may not support efforts to understand what comes *next* in the translational path, how to communicate with stakeholders from different fields, or how to keep their requirements, needs and constraints into account, possibly co-developing the target product. The industrial perspective aims exactly to this direction, while considering the prevailing competitive intensity, the company’s capabilities to undertake all development steps in a specific context, the available or preferred regulatory pathways (e.g., facilitated and accelerated), and the complexities of the emerging local healthcare ecosystem; all things that are normally out of the radar of academic researchers, and for which TPPs provide a template to structure their mapping.

Demonstrating competitive advantage in the biomedical field means having the potential to bring substantial improvements to patients, and to do it safely. For this reason, TPPs (also named Target Patient Value Profiles – TPVP) set the priority to explore what a development asset can really bring to the patient in terms of impact on their medical condition and general quality of life, in comparison to existing products. Expert readers will notice how concretely TPPs help to define *clinical utility* from the earliest development steps, at odds with its late [11] – if not missing [9, 12] – consideration in academic research. Will the technology address a niche or a wider population, maybe with the potential of repurposing for multiple clinical conditions? Will it meet regulatory, health technology and reimbursement requirements? And how, and how much, will patients benefit compared to existing options? TTPs help to understand risk to reward, mitigate adverse effects, potential liabilities and budgets, including the product life cycle, constantly and explicitly pursuing the fundamental endpoint of bringing a well-defined and quantitatively estimated benefit to patients.

Indeed, capturing the very final target of translational research while assessing such a complex and dynamically changing panorama may be beyond the ability as well as the interest of specific academic departments: who are really the stakeholders interested in having academic researchers proceed with such a complex objective in mind, and how could they incentivize this kind of proceeding? Which know-how or infrastructure may support this kind of assessment? Regulators, at the origin of the TPP definition [7, 8], make themselves an inconsistent use of the tool (e.g., its use is not common in European regulatory contexts [10]). The World Health Organization is promulgating its use in the field of dementia [13], after successful use for infectious diseases. Governments award grants to academia and small businesses to fill the regulatory, manufacturing and commercialization vacuum and, to this avail, are increasingly implementing technology transfer offices in academic contexts. These help with launching start-ups, collaborating with already existing companies, or dealing with intellectual property. They have the ideal position to help getting aligned also on translational methodologies, leveraging and importing more of the industrial procedures and good practices.

Dealing with very complex topics that can hardly be tackled by individual groups, academics developed a modus operandi that can be defined “collaborative competition”. In this context, different groups collaborate in solving a common problem, while competing for publishing first. This method is indeed efficient, and may greatly benefit from the use of TPPs, to identify research priorities while limiting research overlaps or gaps. Still, even in this simplified case, identifying the stakeholder interested to incentivize the use of TPPs is as challenging as implementing their use.

### Which way forward

How can the academic community manage to introduce and leverage the benefits of TPPs, while overcoming the competing interests of individual institutes? A concrete way forward requires simple and feasible small steps. First, TPPs may be used to operationalize obvious development steps that multiple institutes are following already, albeit inconsistently: biomarker development provides a typical example [14]. Here, TPPs may bring immediate and concrete harmonization, reducing the still significant gaps, failures and costs [3]. Improving the definition and dissemination of translational methodology, enriching it with tools, methodology and infrastructure imported from or shared with industry may support next steps of increasing complexity. Extending a formal examination of the ecosystem that characterizes academic research and connects it, in synergistic or competitive ways, with industry [15] is also needed to extend into the most challenging requirements of such endeavor. Much work is performed in public-private initiatives, often funded by innovation frameworks like the Innovative Medicine (now Health) Initiative (IMI, IHI) or other grant programs (e.g., PathFinder, InterReg) by the European Commission, or the National Center for Advancing Translational Research funded by the U.S National Institute of Health. Funders themselves may help define and use TPPs incorporating the final aims of their investment: this may at once help researchers perform their work, and funders monitor the proceedings. TPP definition and use may also be supported by regulators themselves, who already offer free consultation and educational opportunities mostly unknown among academics, or by EU-funded services like the European Association for Translational Research (EATRIS), expressly meant to support translational researchers.

Much more work is warranted. For now, explaining the structure and use of TPPs for academia [9, 10], and starting to think to an academic TPP as a simplified version of a commercial TPP may be a first concrete steps to open such perspective.
